# Dataset on *in-silico* investigation on triazole derivatives via molecular modelling approach: A potential glioblastoma inhibitors

**DOI:** 10.1016/j.dib.2020.106703

**Published:** 2020-12-30

**Authors:** Abel Kolawole Oyebamiji, Oluwatumininu Abosede Mutiu, Folake Ayobami Amao, Olubukola Monisola Oyawoye, Temitope A Oyedepo, Babatunde Benjamin Adeleke, Banjo Semire

**Affiliations:** a Department of Basic Sciences, Adeleke University, P.M.B. 250, Ede, Osun State, Nigeria; b Department of Chemical Sciences, Osun State University, Osogbo, Osun State, Nigeria; c Department of Mathematics, Faculty of Science, Adeleke University, P.M.B. 250, Ede, Osun State, Nigeria; d Department of Microbiology, Laboratory of Molecular of Biology, Immunology and Bioinformatics, Adeleke University, P.M.B. 250, Ede, Osun State, Nigeria; e Department of Biochemistry, Adeleke University, P.M.B. 250, Ede, Osun State, Nigeria; f Department of Chemistry, University of Ibadan, Ibadan, Oyo State, Nigeria; g Computational Chemistry Laboratory, Department of Pure and Applied Chemistry, Ladoke Akintola University of Technology, P.M.B. 4000, Ogbomoso, Oyo State, Nigeria

**Keywords:** Triazole, Glioblastoma, Inhibitors, *In-silico*, DFT, QSAR, Docking, ADMET

## Abstract

In this work, ten molecular compounds were optimised using density functional theory (DFT) method via Spartan 14. The obtained descriptors were used to develop quantitative structural activities relationship (QSAR) model using Gretl and Matlab software and the similarity between predicted IC_50_ and observed IC_50_ was investigated. Also, docking study revealed the non-bonding interactions between the studied compounds and the receptor. The molecular interactions between the observed ligands and brain cancer protein (PDB ID: 1q7f) were investigated. Adsorption, distribution, metabolism, excretion and toxicity (ADMET) properties were also investigated.

## Specifications Table

**Table:** 

Subject	Computational Chemistry
Specific subject area	Drug Discovery and Development
Type of data	Figure
	Table
	QSAR model
How data were acquired	Spartan’14, Pymol 1.7.4.4, Autodock tool 1.5.6, AutoVina 1.1.2, Discovery Studio 2017.
Data format	Raw data
Parameters for data collection	B3LYP, 6–31G*, Pymol 1.7.4.4, Discovery studio 2017R, Autodock tool 1.5.6 and Autodock vina 1.1.2.
Description of data collection	The research work started with optimizing the selected compounds using DFT. The obtained descriptors from the optimized compounds were extracted and used to develop QSAR model using MLR and Genetic Algrithm. Also, the developed QSAR model was used to predict the biological activites of new set of triazole based drug-like comounds and further subjected to docking. The results obtained were collected and interpreted.
Data source location	Computational Chemistry Research Laboratory, Department of Pure and Applied Chemistry, Ladoke Akintola University of Technology, P.M.B. 4000, Ogbomoso, Oyo State, Nigeria.
Data accessibility	The observed and calculated data can be accessed with the data article

## Value of the Data

• The data obtained from investigated triazole derivatives in this research will assist scientists to know the molecular descriptors that describe its anti-glioblastoma activity.
• Data in this research will disclose the role of individual molecular descriptors obtained from optimised compounds in the developed QSAR model.
• The obtained binding affinity will reveal the ability of each compound to inhibit brain tumor protein (PDB ID: 1q7f).
• ADMET properties of the observed and proposed molecular compounds were also investigated in order to define the nature of triazole derivatives in receptor.

## 1 Data Description

The 2D structures of the molecules used in this research were shown in Table 1. The observed compounds used in this work were obtained from the research carried out by Ewa et al., (2018) [1]. The compounds with inhibition concentration (IC_50_) of ≤10 µM were selected and subjected to quantum chemical calculation using density functional theory via B3LYP (6–31G∗basis set).

**Table 1:** The Schematic diagram of the observed Triazole derivatives [1].

SN	Molecular Structures	IUPAC Name
1		3-Acetyl-28-propynoylbetulin
		
2		28-Propynoylbetulone
		
3		3-Acetyl-28-[1-(4-*fluorobenzyl*)−1H-1,2,3-*triazol*-4-*yl*]carbonylbetulin
		
4		3-Acetyl-28-(1-ethylacetyl-1H-1,2,3-triazol-4-yl)carbonylbetulin
		
5		3-Acetyl-28-[1-(3-*hydroxypropyl*)−1H-1,2,3-*triazol*-4-*yl*]carbonylbetulin
		
6		2-Amino-3-[4-(3-*acetyl*-28-*betulinylcarbonyl*)−1H-1,2,3-*triazol*-1-*yl*]propanoic acid
		
7		28-[1-(4-F*luorobenzyl*)−1H-1,2,3-*triazol*-4-*yl*]carbonylbetulone
		
8		28-[1-(4-C*yanobenzyl*)−1H-1,2,3-*triazol*-4-*yl*]carbonylbetulone
		
9		28-[1-(3′-D*eoxythymidine*-5′-*yl*)−1H-1,2,3-*triazol*-4-*yl*]carbonylbetulone
		
10		28-[1-(1-D*eoxy*-β-*d*-*glucopyranosyl*)−1H-1,2,3-*triazol*-4-*yl*]carbonylbetulone
		

Thirteen descriptors (Table 2) which describe anti-glioblastoma activities of the investigated triazole derivative were obtained and they were used for further research. The descriptors obtained were highest occupied molecular orbital energy (E_HOMO_), lowest unoccupied molecular orbital (E_LUMO_), band gap (BG), molecular weight (MW), area, volume, polar surface area (PSA), ovality, dipole moment (DM), log P, polarisability (POL), hydrogen bond donor (HBD) and hydrogen bond acceptor (HBA).

**Table 2:** Calculated molecular descriptors with anti-glioblastoma activities.

	E_HOMO_	E_LUMO_	BG	DM	MW	AREA	VOL	PSA	OVA	LOG P	POL	HBD	HBA
1	−6.30	**−2.75**	**3.55**	3.72	536.79	571.38	598.14	40.09	1.66	8.09	89.06	0	2
2	−6.29	−1.49	4.80	3.76	492.74	521.02	551.1	33.37	1.60	8.35	84.95	0	2
3	−6.36	−0.99	5.37	6.72	673.95	706.89	726.39	51.01	1.81	10.23	99.03	0	5
4	−6.33	−1.00	5.33	7.57	651.93	692.8	704.58	71.86	1.81	8.21	97.27	0	6
5	−6.39	−0.96	5.43	7.58	623.92	668.32	681.66	71.04	1.78	8.10	95.39	1	6
6	−6.36	−1.02	5.34	6.29	652.92	682.91	695.04	109.53	1.80	6.86	96.49	1	7
7	−6.25	−1.04	5.21	5.98	643.88	659.42	682.81	59.30	1.76	10.02	95.53	0	5
8	−6.28	−1.92	4.36	4.46	650.90	674.84	697.86	74.64	1.77	9.90	96.95	0	6
9	−6.28	−1.12	5.16	6.23	759.98	760.65	777.32	125.34	1.86	7.12	103.21	2	11
10	−6.26	−1.15	5.11	4.81	697.91	692.16	711.58	138.97	1.80	6.03	97.89	4	10

Table 3 revealed the developed QSAR model (which help to probe into biological activities of triazoles derivatives) from the calculated molecular descriptor obtained from optimised compounds using Gretl software and Matlab [2,3]. The selected descriptors used in developing QSAR model were E_HOMO_, E_LUMO_, Vol, Log P, Pol, PSA and Area and the statistical factors considered for QSAR validation were correlation coefficient (R^2^), adjusted correlation coefficient (Adj.R^2^), P-Value, F-Value and MSE. The calculated value for correlation coefficient (R^2^), Adjusted correlation coefficient (Adj. R^2^), P-Value and F-Value were 0.990, 0.958, *P* < 0.0001, 31.03 and 0.085 as shown in Table 4.

**Table 3:** Calculated QSAR model for the observed triazole derivatives.

Equation	F	P-value	R^2^	Adjusted R^2^	MSE
IC_50_ = −88,509.7 - 513.940(E_HOMO_) + 500.156(E_LUMO_) - 174.603(VOL) + 11.3407(Log P) + 2137.77(POL) + 0.587370(PSA) + 1.01540(AREA)	31.03	*P* < 0.0001	0.990	0.958	0.085

**Table 4:** Assessment for validation of Developed QSAR model.

QSAR model validation parameters	Standard value	Developed QSAR model value	Remark
Correlation coefficient (R^2^)	≥0.5	0.990	Pass
Adjusted Correlation coefficient	≥0.6	0.958	Pass
Confidence interval at 95% confidence level i.e. P-value	<0.05	0.031	Pass

**Fig. 1:**
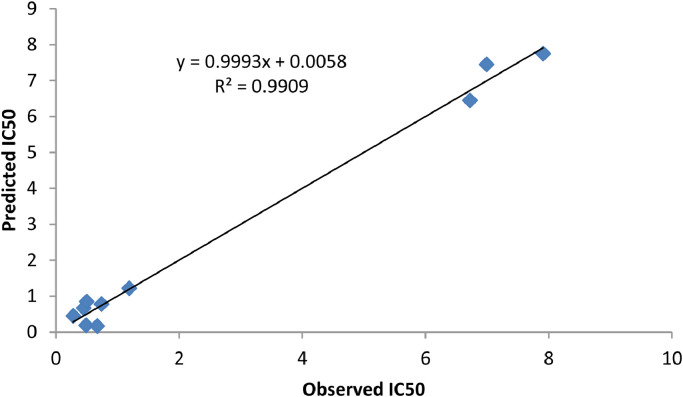
Graphical description of correlation between predicted IC_50_ and Observed IC_50_.

Table 5 showed the calculated inhibition concentration (IC_50_) for the investigated molecular compounds. The correlation between the predicted inhibition efficiency (IC_50_) and observed efficiency (IC_50_) were displayed in Fig. 1. In this work, six (6) molecular compounds were proposed using the developed QSAR model and the inhibition concentration of individual proposed compound was predicted and displayed in Table 6.

**Table 5:** Observed IC_50_ and predicted IC_50_.

	Observed IC_50_	Predicted IC_50_	Residual	Genetic Algorithm (GA)	Residual
1	0.67	0.45	0.217310	0.616731	0.053269
2	0.19	0.49	−0.307571	0.136731	0.053269
3	0.85	0.50	0.342669	0.816023	0.033977
4	0.78	0.74	0.0302183	0.746023	0.033977
5	7.75	7.91	−0.161626	7.716023	0.033977
6	1.22	1.19	0.0226067	1.186023	0.033977
7	0.45	0.28	0.167543	0.416023	0.033977
8	6.45	6.72	−0.270293	6.416796	0.033204
9	0.17	0.67	−0.500773	0.137068	0.032932
10	7.45	6.99	0.459915	7.417068	0.032932

**Table 6:** Schematics structures of the proposed compounds with the inhibition concentration.


	R_1_	R_2_	IC_50_
1	OCH_3_	CH_3_	−13.54
2	OC_2_H_5_	CH_3_	−9.60
3	NH_2_	CH_3_	−16.79
4	CH_3_	CH_3_	−7.72
5	CH_2_F	CH_3_	−27.30
6	CHF_2_	CH_3_	−32.98

Also, Table 7 showed four molecular compounds (**2, 7, 9** and **10)** with −9.5 kcal/mol, **−**11.2 kcal/mol**,** −10.0 kcal/mol, and −9.4 kcal/mol respectively. The selected compounds were subjected to ADMET study using admetSAR server and the factor considered were based on adsorption, distribution, metabolism, excretion and toxicity of the investigated ligands. The obtained ADMET values were compared to the standard compound used (Carmustine).

**Table 7:** Obtained calculated ADMET Properties.

	Compound 2		Compound 7		Compound 8		Compound 9		Compound 10		Carmustine	
Mode	Result	Probability	Result	Probability	Result	Probability	Result	Probability	Result	Probability	Result	Probability
Blood-Brain Barrier	BBB+	0.9079	BBB+	0.5892	BBB+	0.5456	BBB-	0.8875	BBB-	0.9564	BBB+	0.9533
Human Intestinal Absorption	HIA+	0.9900	HIA+	0.9962	HIA+	0.9947	HIA+	0.9430	HIA+	0.6024	HIA+	1.0000
Caco-2 Permeability	Caco2+	0.6469	Caco2-	0.5649	Caco2-	0.5831	Caco2-	0.6729	Caco2-	0.6997	Caco2-	0.5621
P-glycoprotein Substrate	Substrate	0.6290	Substrate	0.8512	Substrate	0.8125	Substrate	0.8454	Substrate	0.8269	Non-Substrate	0.7552
P-glycoprotein Inhibitor	Inhibitor	0.9030	Inhibitor	0.9365	Inhibitor	0.9495	Inhibitor	0.6937	Inhibitor	0.7651	Non-inhibitor	0.7970
	Inhibitor	0.7882	Inhibitor	0.8550	Inhibitor	0.9370	Inhibitor	0.5500	Inhibitor	0.5758	Non-inhibitor	0.8778
Renal Organic Cation Transporter	Non-inhibitor	0.7387	Non-inhibitor	0.5700	Non-inhibitor	0.5301	Non-inhibitor	0.8682	Non-inhibitor	0.9224	Non-inhibitor	0.8177
Subcellular localization	Mitochondria	0.8457	Mitochondria	0.6253	Mitochondria	0.6304	Mitochondria	0.5907	Mitochondria	0.4545	Mitochondria	0.7342
CYP450 2C9 Substrate	Non-substrate	0.8652	Non-substrate	0.8442	Non-substrate	0.8067	Non-substrate	0.7938	Non-substrate	0.7848	Non-substrate	0.7656
CYP450 2D6 Substrate	Non-substrate	0.9104	Non-substrate	0.8250	Non-substrate	0.8210	Non-substrate	0.8343	Non-substrate	0.8260	Non-substrate	0.8491
CYP450 3A4 Substrate	Substrate	0.7739	Substrate	0.6987	Substrate	0.7062	Substrate	0.7076	Substrate	0.6901	Non-substrate	0.6720
CYP450 1A2 Inhibitor	Non-inhibitor	0.8848	Non-inhibitor	0.7102	Non-inhibitor	0.7503	Non-inhibitor	0.8277	Non-inhibitor	0.7723	Non-inhibitor	0.9045
CYP450 2C9 Inhibitor	Non-inhibitor	0.6679	Non-inhibitor	0.5957	Non-inhibitor	0.6198	Non-inhibitor	0.6658	Non-inhibitor	0.7210	Non-inhibitor	0.9070
CYP450 2D6 Inhibitor	Non-inhibitor	0.9248	Non-inhibitor	0.8454	Non-inhibitor	0.8666	Non-inhibitor	0.8863	Non-inhibitor	0.9026	Non-inhibitor	0.9231
CYP450 2C19 Inhibitor	Inhibitor	0.5296	Inhibitor	0.5581	Non-inhibitor	0.5391	Non-inhibitor	0.7017	Non-inhibitor	0.7290	Non-inhibitor	0.9025
CYP450 3A4 Inhibitor	Non-inhibitor	0.6446	Inhibitor	0.7561	Inhibitor	0.7227	Inhibitor	0.9283	Inhibitor	0.6378	Non-inhibitor	0.9031
CYP Inhibitory Promiscuity	Low CYP Inhibitory Promiscuity	0.5796	High CYP Inhibitory Promiscuity	0.8442	High CYP Inhibitory Promiscuity	0.7893	High CYP Inhibitory Promiscuity	0.6255	High CYP Inhibitory Promiscuity	0.5093	Low CYP Inhibitory Promiscuity	0.9131
Human Ether-a-go-go-Related Gene Inhibition	Weak inhibitor	0.9102	Weak inhibitor	0.6081	Weak inhibitor	0.5223	Weak inhibitor	0.7555	Weak inhibitor	0.9532	Strong inhibitor	0.7278
	Non-inhibitor	0.7874	Non-inhibitor	0.5632	Non-inhibitor	0.7159	Non-inhibitor	0.7011	Non-inhibitor	0.5634	Non-inhibitor	0.9190
AMES Toxicity	Non AMES toxic	0.8923	Non AMES toxic	0.5299	Non AMES toxic	0.5228	Non AMES toxic	0.5140	Non AMES toxic	0.5885	AMES toxic	0.9577
Carcinogens	Non-carcinogens	0.8769	Non-carcinogens	0.8221	Non-carcinogens	0.8539	Non-carcinogens	0.7862	Non-carcinogens	0.9015	Carcinogens	0.6880
Fish Toxicity	High FHMT	0.9996	High FHMT	1.0000	High FHMT	0.9999	High FHMT	0.9998	High FHMT	0.9999	High FHMT	0.6546
Tetrahymena Pyriformis Toxicity	High TPT	0.9996	High TPT	0.9968	High TPT	0.9931	High TPT	0.9874	High TPT	0.9924	High TPT	0.9857
Honey Bee Toxicity	High HBT	0.8781	Low HBT	0.6610	Low HBT	0.5742	Low HBT	0.6662	Low HBT	0.6038	Low HBT	0.7045
Biodegradation	Not ready biodegradable	0.9800	Not ready biodegradable	1.0000	Not ready biodegradable	1.0000	Not ready biodegradable	0.9870	Not ready biodegradable	0.9803	Not ready biodegradable	0.5596

**Fig. 2:**
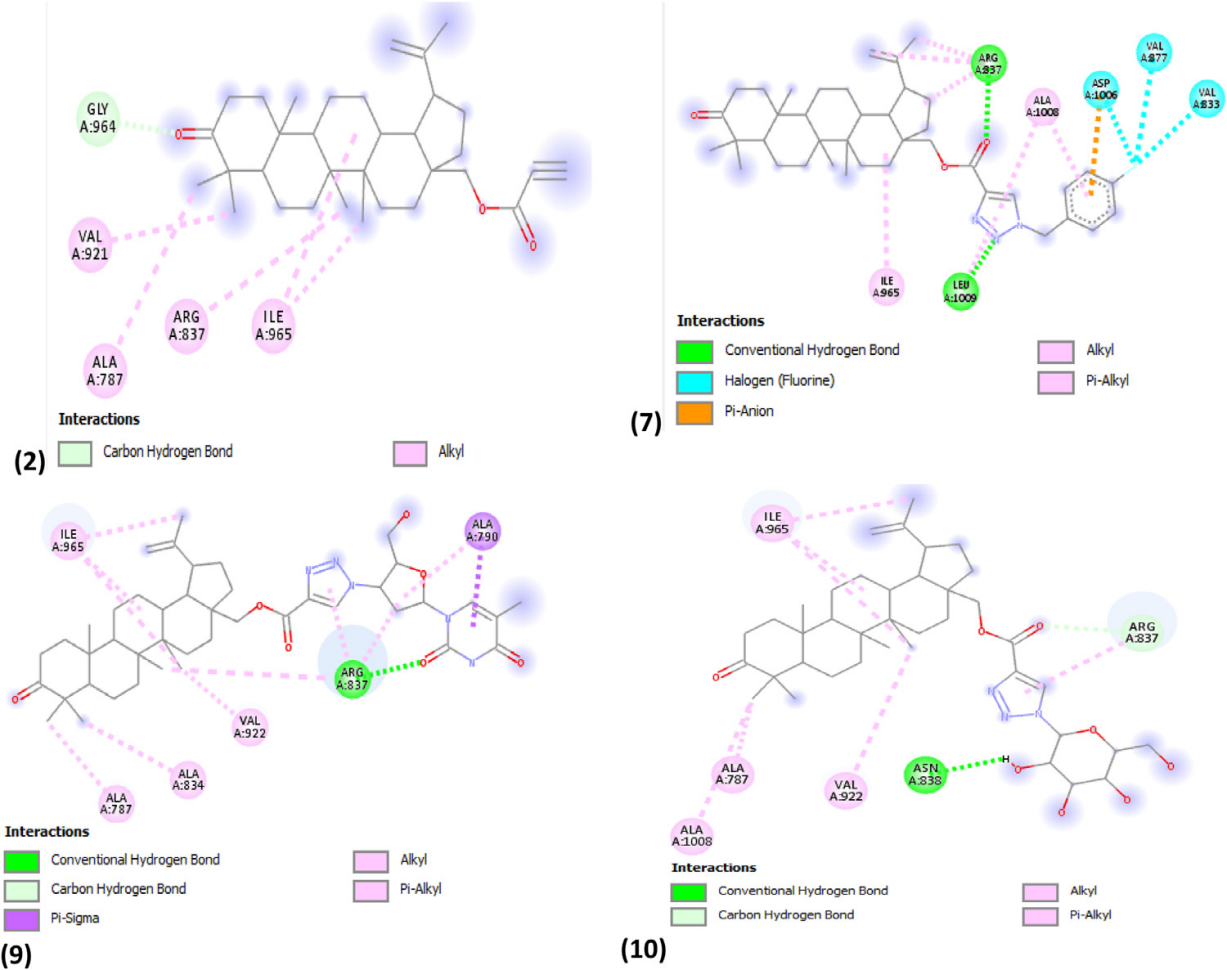
2D structures of brain tumor protein (PDB ID: 1q7f) and compound **2, 7, 8, 9** and **10** respectively.

The calculated molecular interaction observed between the optimised triazole derivatives and brain tumor protein (PDB ID: 1q7f) [4] were reported in Table 8. The binding affinity calculated for each complex was −8.4 kcal/mol, −9.5 kcal/mol, −8.6 kcal/mol, −8.8 kcal/mol, −8.5 kcal/mol, −8.1 kcal/mol, −11.2 kcal/mol, −9.0 kcal/mol, −10.0 kcal/mol and −9.4 kcal/mol for compound **1–10** and the interaction between the observed complexes were shown in Fig. 2.

**Table 8:** Scoring and residues involved in the interaction between the studied complex.

	Scoring (kcal/mol)	Residues involved in the interactions	Types of Non-bonding interaction involved
**1**	−8.4	VAL-835, VAL-788, VAL-921, LEU-1sss009, ILE-965	Conventional Hydrogen Bond, Alkyl
**2**	−9.5	GLY-964, VAL-921, ALA-787, ARG-837, ILE-965	Carbon Hydrogen Bond, Alkyl
**3**	−8.6	ILE-965, VAL-1007, ALA-787, ALA-834, VAL-922, ARG-837	Carbon Hydrogen Bond, Alkyl
**4**	−8.8	ASP-924, ARG-837, ALA-834, VAL-877	Conventional Hydrogen Bond, Carbon Hydrogen Bond, Pi-Alkyl, Alkyl
**5**	−8.5	ASP-924, ILE-965, VAL-950, THR-878, LEU-1009, ALA-1008, ARG-837	Conventional Hydrogen Bond, Carbon Hydrogen Bond, Pi-Alkyl, Alkyl
**6**	−8.1	THR-986, GLN-987, GLY-969, ASN-968, ILE-965, VAL-922, ARG-837, VAL-835	Conventional Hydrogen Bond, Carbon Hydrogen Bond, Alkyl
**7**	**−11.2**	ARG-837, ALA-1008, ASP-1006, VAL-877, VAL-833, LEU-1009, ILE-965	Conventional Hydrogen Bond, Halogen(Fluorine), Pi-Anion, Pi-Alkyl, Alkyl
**8**	−9.0	ILE-965, GLY-969, VAL-922, ARG-837, ALA-1008, ALA-787	Carbon Hydrogen Bond, Alkyl
**9**	−10.0	ILE-965, ALA-787, ALA-834, VAL-922, ARG-837, ALA-790	Conventional Hydrogen Bond, Carbon Hydrogen Bond, Pi-Alkyl, Pi-Sigma, Alkyl
**10**	−9.4	ILE-965, ALA-787, ALA-1008, VAL-922, ASN-838, ARG-837	Conventional Hydrogen Bond, Carbon Hydrogen Bond, Pi-Alkyl, Alkyl
**Carmustine**	−5.0	VAL-835, ARG-837, VAL-879	Conventional Hydrogen Bond

**Proposed Compounds**			

**P1**	−8.6	PHE-916, ILE-961, TYR-959	Alkyl, Pi-Alkyl
**P2**	−8.7	PHE-916, ILE-961, TYR-959	Alkyl, Pi-Alkyl
**P3**	−8.5	VAL-788, VAL-835, ARG-837, ILE-965	Conventional Hydrogen Bond, Unfavourable Donor-Donor, Alkyl
**P4**	−8.7	PHE-916, ILE-961, TYR-959	Alkyl, Pi-Alkyl
**P5**	−8.6	PHE-1005, PHE-916, ILE-961, TYR-959	Alkyl, Pi-Alkyl
**P6**	−8.9	ARG-837, VAL-788, ALA-787, VAL-835, ALA-834, ILE-965, VAL-921	Conventional Hydrogen Bond, Halogen, Alkyl

## 2 Design, Materials and Methods

The studied triazole derivatives (Table 1) were drawn using ChemDraw Ultra 8.0 and were optimised using Spartan 14 [5]. The optimization was accomplished using B3LYP with 6–31 G* as basis set which produce descriptors that were used for further investigation. The selected calculated descriptors obtained from the optimised compounds were used to build robust QSAR model in order to relate the biological activity of the studied compounds to the calculated molecular parameters [6]. This was achieved using mathematical methods (multiple linear regression method) via Gretl 1.9.8. The observed inhibition concentration (IC_50_) served as dependent variable while the calculated descriptors served as independent variables; thus, the QSAR model was developed. Several factors such as correlation coefficient (R^2^), P-Value, F-value were considered to know the level of efficiency of the developed QSAR model. More so, validation of the developed QSAR model was implemented by observing some mathematical factors (cross validation correlation coefficient (C.V R^2^), adjusted correlation coefficient) which could be calculated using Eq. (1) and 2 [7].

(1) $$C.VR^{2}=1-\;\frac{\sum {\left(Y_{obs}-\;Y_{cal}\right)}^{2}}{\sum {\left(Y_{obs}-\;{\bar{Y}}_{obs}\right)}^{2}}$$

(2) $$R_{a}^{2}=\frac{\left(N-1\right)\times \;R^{2}-P}{N-1-P}$$

Absorption, Distribution, Metabolism, Excretion and the Toxicity properties of the studied triazole derivatives were done via online software (admetSAR) (http://lmmd.ecust.edu.cn/admetsar1) [8]. The factors considered were Blood Brain Barrier, Caco-2 cell permeability, Human Intestinal Absorption, Ames test. Also, four software (Pymol (for treating downloaded protein), Autodock Tool (for locating binding site in the downloaded protein and for converting ligand and receptor to.pdbqt format from.pdb format), Auto dock vina (for docking calculation) and discovery studio (for viewing the non-bonding interaction between the docked complexes) were used to accomplish docking study between triazole derivative and brain tumor protein (PDB ID: 1q7f). The observed grid box was as follows: center (*X* = 12.534, *Y* = 23.847, *Z* = 40.848) and size (*X* = 68, *Y* = 64, *Z* = 72) as well as the spacing was set to be 1.00 Å.

## Ethics Statement

Not Applicable.

## CRediT Author Statement

Abel Kolawole **OYEBAMIJI:** Conceptualization, Methodology, Writing- Original draft preparation; Oluwatumininu Abosede **MUTIU**: Software; Folake Ayobami **AMAO**, Data curation; Olubukola Monisola **OYAWOYE**: Writing- Reviewing and Editing; Temitope A **OYEDEPO:** Writing- Reviewing and Editing; Babatunde Benjamin **ADELEKE**: Visualization; Banjo **SEMIRE:** Supervision, Software, Validation.

## Declaration of Competing Interest

The authors declare that they have no conflict of interest.
